# Genetic Dissection of Three Major Quantitative Trait Loci for Spike Compactness and Length in Bread Wheat (*Triticum aestivum* L.)

**DOI:** 10.3389/fpls.2022.882655

**Published:** 2022-05-23

**Authors:** Qin Yu, Bo Feng, Zhibin Xu, Xiaoli Fan, Qiang Zhou, Guangsi Ji, Simin Liao, Ping Gao, Tao Wang

**Affiliations:** ^1^Chengdu Institute of Biology, Chinese Academy of Sciences, Chengdu, China; ^2^College of Life Sciences, Sichuan University, Chengdu, China; ^3^University of Chinese Academy of Sciences, Beijing, China; ^4^Innovative Academy of Seed Design, Chinese Academy of Sciences, Beijing, China

**Keywords:** spike compactness, spike length, BSE-Seq, QTL, pyramiding, wheat

## Abstract

Spike compactness (SC) and length (SL) are the components of spike morphology and are strongly related to grain yield in wheat (*Triticum aestivum* L.). To investigate quantitative trait loci (QTL) associated with SC and SL, a recombinant inbred lines (RIL) population derived from the cross of Bailangmai (BLM, a Tibet landrace) and Chuanyu 20 (CY20, an improved variety) was employed in six environments. Three genomic regions responsible for SC and SL traits were identified on chromosomes 2A and 2D using bulked segregant exome sequencing (BSE-Seq). By constructing genetic maps, six major QTL were repeatedly detected in more than four environments and the best linear unbiased estimation (BLUE) datasets, explaining 7.00–28.56% of the phenotypic variation and the logarithm of the odd (LOD) score varying from 2.50 to 13.22. They were co-located on three loci, designed as *QSc/Sl.cib-2AS*, *QSc/Sl.cib-2AL*, and *QSc/Sl.cib-2D*, respectively. Based on the flanking markers, their interactions and effects on the corresponding trait and other agronomic traits were also analyzed. Comparison analysis showed that *QSc/Sl.cib-2AS* and *QSc/Sl.cib-2AL* were possibly two novel loci for SC and SL. *QSc/Sl.cib-2AS* and *QSc/Sl.cib-2D* showed pleiotropic effects on plant height and grain morphology, while *QSc/Sl.cib-2AL* showed effects on spikelet number per spike (SNS) and grain width (GW). Based on the gene annotation, orthologous search, and spatiotemporal expression patterns of genes, *TraesCS2A03G0410600* and *TraesCS2A03G0422300* for *QSc/Sl.cib-2AS*, and *TraesCS2D03G1129300* and *TraesCS2D03G1131500* for *QSc/Sl.cib-2D* were considered as potential candidate genes, respectively. These results will be useful for fine mapping and developing new varieties with high yield in the future.

## Introduction

Hexaploidy wheat (*Triticum aestivum* L.), one of the most widely planted food crops, provides approximately 20% of the dietary calories in food products consumed worldwide. To satisfy the demand of the rapidly expanding global population, wheat production needs annual growth of approximately 2% during the next three decades, but the actual growth rates are generally not more than 1% each year (Gao et al., 2016a; Cao et al., 2020; Saini et al., 2022). Therefore, genetic improvement in yield potential combined with a better field management system may be a feasible strategy to increase the grain yield (Yang et al., 2020). The spike is the reproductive organ of the wheat plant and is an essential component to produce and hold grains (Faris et al., 2014). The morphological traits of a spike, including spike compactness (SC), spike length (SL), and spikelet number per spike (SNS), have been found to be greatly associated with grain yield in previous studies (Chai et al., 2019; Wolde et al., 2019; Li et al., 2021a; You et al., 2021). Therefore, the identification of major and stable quantitative trait loci (QTL)/genes for SC and SL is an efficient strategy to cultivate and domesticate high-yield varieties with the ideal plant architecture in wheat for breeders (Ma et al., 2007; Faris et al., 2014).

Three major genes are associated with spike morphology: *Compactum* (*C*), *Sphaerococcum* (*S*), and *Q* (Fan et al., 2019). The *C* gene, anchored on the long arm of chromosome 2D near the centromere, shows pleiotropic effects on spike compactness, grain size and shape, and the grain number per spike (Johnson et al., 2007). The *S* gene is located on chromosome 3D and defines a loose spike and semispherical grains (Faris et al., 2014). The *Q* gene, located on the long arm of chromosome 5A, is a member of the AP2 class of transcription factors and shows pleiotropic effects on rachis fragility, glume shape and tenacity, spike length, plant height, and heading date (Fa Ris and Gill, 2002; Faris et al., 2003; Simons et al., 2005; Xu et al., 2018). Meanwhile, three groups of genes, *vernalization* (*Vrn*, vernalization requirement), *photoperiod* (*Ppd*, photoperiod sensitivity), and *earliness per se* (*Eps*), control life-cycle duration which affects the SC and SL in wheat (Kato and Yamagata, 1988; Wang et al., 2014). *Vrn* and *Ppd* genes commonly regulated the transition from the vegetative to reproductive growth phase, thus affecting heading, flowering, and maturity time (Laurie et al., 1995; Kane et al., 2005; Dubcovsky et al., 2006; Hemming et al., 2008). The *earliness per se* gene *Eps-A*^m^*1* from diploid wheat *Triticum monococcum* affects the heading time, spike development, and spikelet number (Faricelli et al., 2016). However, *Earliness per se 3* (*Eps-3*) may have functions in the initiation of spikelet meristem in wheat (Li et al., 2018). Furthermore, MADS-box genes, *FUL2* and *FUL3*, play critical and redundant roles in the development of spikelets and spikes and also affect flowering time and plant height in wheat (Li et al., 2019a). Moreover, *Rht5* (Chen et al., 2018a), *Rht8* (Kowalski et al., 2016), *Rht22* (Peng et al., 2011), *Rht24* (Tian et al., 2017), and *Rht25* (Mo et al., 2018) are GA-responsive and all exhibit pleiotropic effects on SC and SL in addition to reducing height. *TasgD1*, a grain-shaped gene, is identified by the positional cloning approach and shows pleiotropic effects on spike morphological traits (Cheng et al., 2020).

Similar to other yield-related traits, SC and SL are complex quantitative traits that are influenced by the interaction between genetic and environmental factors. QTL for SC and SL were found to be distributed on all the 21 chromosomes, which was based on genetic linkage analysis in bi-parental genetic populations and genome-wide association study (GWAS) over the past decades (Kumar et al., 2006; Marza et al., 2006; Deng et al., 2011; Cui et al., 2012, 2014; Jia et al., 2013; Patil et al., 2013; Katkout et al., 2014; Lee et al., 2014; Liu et al., 2014; Fan et al., 2015; Li et al., 2016, 2019b; Zhai et al., 2016; Chen et al., 2017; Sheoran et al., 2019). However, only a few of them were identified to be stable in multiple environments and were validated in different genetic backgrounds, which restrict the favorable allele usage in wheat breeding programs.

In the present study, a recombinant inbred lines (RIL) population obtained by the crossing of Bailangmai (BLM) and Chuanyu 20 (CY20) was utilized for the dissection of the genetic determinants of SC and SL. The exome capture sequencing of bulked segregant analysis (BSE-Seq) was used to identify the genomic regions that are responsible for the two traits and polymorphic SNPs and InDels for marker development, which was further used to construct the genetic map. Herein, the objectives of this study were to: (i) phenotypically evaluate the performance of SC and SL in the BC20 population in multiple environments; (ii) dissect the genomic regions controlling SC and SL by BSE-Seq; (iii) identify stable and major QTL, and analyze their effects and interactions; and (iv) predict candidate genes for gene cloning.

## Materials and Methods

### Plant Materials and Field Experiments

A RIL (F_10_) population with 182 lines was developed from an F_2_ population derived from the cross of Bailangmai × Chuanyu 20 (BLM/CY20, BC20) by the single-seed descent method. BLM is a Tibet landrace that can resist extreme stress conditions, such as cold and drought, and shows strong tillering capacity. CY20 is an improved variety with high yield.

The parents and lines of the BC20 population were grown at a conventional sowing time across two different sites over three growing seasons (that is, six environments), including Shuangliu (103°52′E, 30°34′N) in 2019–2020 (E1), Shifang (104°11′E, 31°6′N) in 2019–2020 (E2), Shifang in 2018–2019 (E3), Shuangliu in 2018–2019 (E4), Shuangliu in 2017–2018 (E5), and Shifang in 2017–2018 (E6). A randomized complete block design was adopted for all of the trials. Each line was represented by a single 1-m row plot with a sowing rate of 11 seeds and a spacing of 20 cm between the rows. Two replicates were used in this study. Field management was performed according to the local standard practices for wheat production.

### Phenotypic Evaluation and Statistical Analysis

After harvesting the whole plants at maturity, eight representative plants of each line and parents were selected randomly to measure the phenotype of agronomic traits, including plant height (PHT), productive tiller number (PTN), spike length (SL), spikelet number per spike (SNS), spike compactness (SC), grain number per spike (GNS), 1,000-grain weight (TGW), grain length (GL), grain width (GW) and grain roundness (GR). PHT was measured from the plant base to the tip of the main spike excluding the awns. SL was the length from the base of the stalk to the top of the main spike excluding the awns. SNS was determined by counting the number of spikelets in the main spike. SC was calculated by dividing SNS by SL. The traits of grains were evaluated by using the software SC-G (WSeen, Hangzhou, China).

Basic statistical analyses, frequency distribution, Pearson’s correlations analyses among different traits, and Student’s *t*-test (*P* < 0.05) for evaluating the significance of difference were performed using the software SPSS 20 (IBM SPSS, Armonk, NY, United States). The best linear unbiased evaluation (BLUE), combined QTL detection and effect analyses were performed using QTL IciMapping 4.2^1^. The broad-sense hereditary capacity (*H*^2^) of each trait was estimated according to the method described by Smith et al. (1998) and Muqaddasi et al. (2019).

### Bulked Segregant Analysis and Exome Sequencing

The genomic DNA of each line and the parents was isolated from 14-day-old seedlings using the modified cetyl trimethyl ammonium bromide (CTAB) method, followed by RNase-A digestion. The integrity of DNA was checked and confirmed on the agarose gels, and the concentration of DNA was calculated using a spectrophotometer. Based on the phenotypic values obtained in six environments and the BLUE datasets, all the lines in each environment were arranged in order from small to large separately. The lines within two tails (20%) in the BLUE datasets and simultaneously in at least five of the six environments were selected to bulk pools. The genomic DNA was bulked into four extreme pools by collecting equal quantities (1 μg) of DNA from 30 individuals with extremely high SC value (SC-H), low SC value (SC-L), high SL value (SL-H), and low SL (SL-L) value in the BC20 population, respectively. Preparation and resequencing of six DNA libraries from BLM, CY20, SC-H, SC-L, SL-H, and SL-L pools were conducted by Bioacme Biotechnology Co., Ltd. (Wuhan, China^2^).

Clean data, without the reads containing sequencing adapters, low-quality bases, or undetected bases, were used for the subsequent analysis (Chen et al., 2018b). The alignment tool BWA (alignment of burrows-wheeler) was used to align the clean data to the Chinese Spring (CS) reference genome sequence (RefSeq) v1.0 released by the International Wheat Genome Sequencing Consortium (IWGSC) (Li, 2013). BCFtools were used to detect and extract the single nucleotide polymorphism (SNP) and InDel sites (Danecek et al., 2016). The SNP and InDel sites were annotated using ANNOVAR, which mainly included different regions of the genome and different types of exon regions (Kai et al., 2010). Euclidean distance (ED) and SNP-index methods were used to screen the SNP and InDel sites with significant differences between the progeny mixed pools of two traits in this study (Abe et al., 2012; Hill et al., 2013; Takagi et al., 2013; Li et al., 2020).

Axiom^®^Wheat 660K Genotyping Arrays, a high-density SNP chip for wheat, was also used for detecting the polymorphic SNPs between the two parents and was carried out by China Golden Marker (Beijing, China).

### Development of Molecular Markers

Based on the BSE-Seq analysis and wheat 660K SNP assay, SNPs and InDels in the target genomic regions between BLM and CY20 were screened to design Kompetitive Allele-specific PCR (KASP) markers and simple sequence repeat (SSR) markers using the online websites of Triticeae Multi-omics Center^3^ and Galaxy^4^, respectively. In the primer designing of KASP markers, the FAM and HEX probe sequences were added to the 5′ terminal of the primers. The KASP assays were performed in QuantStudio™ 3 Real-Time PCR System designed by Thermo Fisher Scientific with the reaction mixture containing 5 μl of 2 × master mix, 0.8 μl of primer mix, 3 μl of ddH_2_O, and 2 μl of DNA template (50–100 ng/μl). Touchdown PCR conditions were hot-start activation at 95°C for 15 min, followed by a touchdown phase of 10 cycles (95°C for 20 s, initial touchdown at 61°C, and then decreased by 0.6°C per cycle for 1 min), and finally 26 cycles of regular PCR (95°C for 20 s; 55°C for 40 s). The final fluorescence was read at 35°C for 30 s. If the clustering was not significant, more cycling and resting steps were required at the following conditions: 94°C for 20 s, followed by 55°C for 40 s (2–5 cycles per step).

The regular PCR for SSR primers was conducted in a 20 μl reaction volume consisting of 10 μl of 2 × Taq Master Mix, 1 μl of primer mixture (10 μM), 0.5 μl of DNA template (∼100 ng/μl), and 8.5 μl of ddH_2_O. The conditions followed for SSR PCR were similar to those followed for PCR. Polyacrylamide gel (8%) electrophoresis was used for separating the amplification products.

### Genetic Map Construction and Quantitative Trait Loci Detection

JoinMap 4.1 and QTL IciMapping 4.2 were used for genetic map construction and QTL detection in this study separately (Meng et al., 2015). First, the markers that were redundant and had a missing rate >20% were discarded. Then, the function of “Population” in JoinMap 4.1 was used to create groups with LOD score values ranging from 2 to 10. Finally, the Kosambi mapping function was used to order the markers with the parameters being set as LOD ≥ 5 and round = 3 in JoinMap 4.1. QTL detection in each environment was performed by IciMapping 4.2 based on the Biparental Populations (BIP) module with the inclusive composite interval mapping (ICIM), and a test of 1,000 permutations was performed to identify the LOD threshold that corresponded to a genome-wide false discovery rate (FDR) of 5% (*P* < 0.05). The missing phenotype was represented as −100 in the QTL analysis (Li et al., 2021c). The QTL with overlapping intervals were considered to be equivalent and named according to the rules of International Rules of Genetic Nomenclature^5^.

### Prediction of Candidate Genes

The physical positions of the markers, derived from the genetic map, were converted from IWGSC RefSeq v1.0 to RefSeq v2.1 using the tools of GetSequence and BLAST of the WheatOmics (see Text Footnote 3) (Zhu et al., 2021). Genes between the flanking markers were extracted using the Interval Tools of the WheatOmics. The annotations and functions of the given gene were analyzed using UniProt^6^. The expression pattern analysis of candidate genes was performed by using GeneExpression of WheatOmics. The expression data of each gene in different tissues were normalized using the ZeroToOne method and then presented in the HeatMap drawn by TBtools (Chen et al., 2020a). Meanwhile, to analyze the potential candidate genes, non synonymous SNPs in the exon region of genes in the target regions were collected using the BSE-Seq result.

## Results

### Phenotypic Variation and Correlation Analysis

**FIGURE 1:**
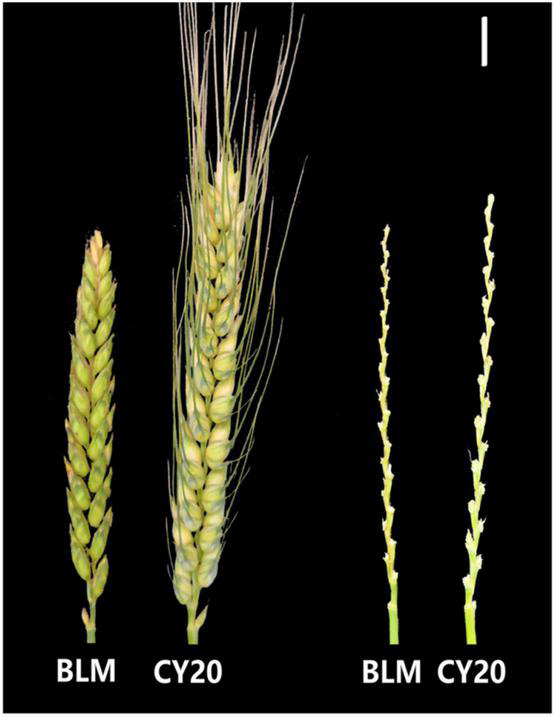
Spike morphology of the parental lines BLM and CY20 growing in the Shuangliu region (2019–2020 growing season). The bar represents 1 cm.

**FIGURE 2:**
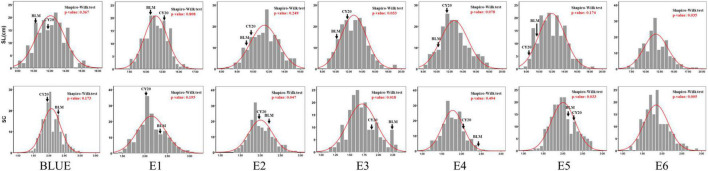
Frequency distribution of BC20 lines for spikelet compactness (SC) and spike length (SL) in six environments and the BLUE datasets.

Significant differences in SC and SL between BLM and CY20 were detected in multiple environments and the BLUE datasets (Figure 1 and Table 1). As observed, CY20 had a longer and less compact spike than BLM. In the BC20 population, both SC and SL showed wide and significant variations, with SL ranging from 7.12 to 18.33 cm and SC ranging from 1.13 to 3.04 (Table 1). According to the perspective of skewness, kurtosis, and the pattern of continuous distribution for SC and SL, both traits showed typical normal distribution and obvious bidirectional transgressive segregation appearance in multiple environments and the BLUE datasets, indicating that they are common quantitative traits controlled by multiple genes (Figure 2 and Table 1). The broad heritability (*H*^2^) of SC and SL was 93.46% and 93.49%, and the coefficient of variation was 13.89–16.47% and 14.53–17.81% for SC and SL, respectively. These results indicated that both SC and SL were environmentally stable and mostly controlled by genetic factors (Table 1). Moreover, the results of the phenotypic evaluation, combined analyses of variance (ANOVA), and broad-sense heritability (*H*^2^) estimates for other agronomic traits, such as PHT, PTN, SNS, GNS, TGW, GL, GW, and GR, showed typical normal distribution and obvious bidirectional transgressive segregation appearance in the BLUE datasets, which was similar to those observed for SC and SL (Table 1 and Supplementary Tables 1, 2). Furthermore, significant positive correlations for SC and SL were also detected between six different environments in the BC20 population, with the Pearson’s correlation (*r*) value of 0.55–0.92 and 0.64–0.89 for SC and SL, respectively (Supplementary Table 3).

**TABLE 1:** Phenotypic variation and heritability (*H*^2^) of spike compactness (SC) and spike length (SL) for parents and BC20 lines in different environments and the BLUE datasets.

Traits	Environment	Parents		BC20 lines					
		BLM	CY20	Range	Mean ± *SD*	CV (%)	Skewness	Keratosis	*H* ^2^
SL	BLUE	10.58	11.88*	8.4–16.89	12.10 ± 1.76	14.53	0.14	−0.35	93.49
	E1	11.25	12.16*	7.59–16.24	11.52 ± 1.74	15.09	0.04	−0.39	
	E2	9.3	9.89*	6.95–15.49	11.44 ± 1.77	15.51	0.04	−0.45	
	E3	10.2	11.6*	9–19.14	12.75 ± 1.92	15.07	0.38	0.13	
	E4	10.47	11.7*	8–18	12.71 ± 2.09	16.42	0.3	−0.26	
	E5	9.33	8.17	7.67–18.33	12.01 ± 2.08	17.36	0.31	−0.38	
	E6	−	−	7.12–18.75	11.81 ± 2.1	17.81	0.43	0.32	
SC	BLUE	2.3	1.97**	1.40–2.97	2.08 ± 0.29	13.89	0.42	0.02	93.46
	E1	2.32	2.04*	1.45–3.04	2.15 ± 0.31	14.39	0.41	−0.03	
	E2	2.25	2.05*	1.34–2.9	2.01 ± 0.29	14.58	0.47	0.02	
	E3	2.24	1.86**	1.16–2.33	1.71 ± 0.25	14.61	0.41	−0.19	
	E4	2.45	2.03**	1.13–2.52	1.78 ± 0.27	15.01	0.15	−0.21	
	E5	2.1	2.26	1.31–2.74	2.01 ± 0.3	15.16	0.4	−0.38	
	E6	−	−	1.23–2.88	1.85 ± 0.3	16.47	0.62	0.24	

*BLUE, best linear unbiased estimation; CV, coefficient of variation; H^2^, broad-sense heritability.*

* and ** represent significance at P < 0.05 and P < 0.01, respectively.

**FIGURE 3:**
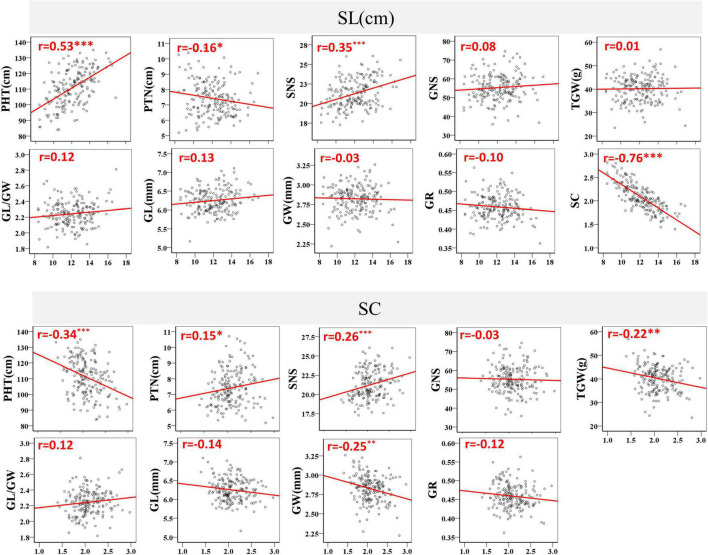
Coefficients of pair-wise Pearson’s correlations between spike compactness (SC), spike length (SL), plant height (PHT), productive tiller number (PTN), spikelet number per spike (SNS), grain number per spike (GNS), thousand-grain weight (TGW), grain width (GW), grain length (GL), GL/GW ratio, and grain roundness (GR) in the BC20 population. *, ^**^, and ^***^ represent significance at *P* < 0.05, *P* < 0.01, and *P* < 0.001, respectively.

The coefficients of pair wise Pearson’s correlations between SC, SL and other yield-related traits were determined to evaluate trait correlations using the BLUE datasets. A significant and negative correlation was detected between SC and SL, with *r* = −0.76 and *P* < 0.001 (Figure 3). SL was strongly positively correlated with PHT and weakly negatively correlated with PTN. In contrast, SC was significantly negatively correlated with PHT and positively correlated with PTN. SNS was the only trait that was significantly positively correlated with SL and SC, with *P* < 0.001 (Figure 3). Moreover, SC negatively correlated with the grain traits TGW (*r* = −0.22) and GW (*r* = −0.25) at a significance level of *P* < 0.01. However, no significant correlation was observed between SC, SL and GNS, GL/GW, GL, GR, and between SL and TGW, GW (Figure 3).

### The Exome Capture Sequencing of Bulked Segregant Analysis for Spike Compactness and Spike Length

The exome capture sequencing of bulked segregation (BSE-Seq) analysis was performed to identify the genomic regions for SC and SL, and the results were compared with the Chinese Spring (CS) reference genome v1.0 by IWGSC. Then, we invented the final physical regions from CS RefSeq v1.0 into CS RefSeq v2.1.

The total number of clean reads after filtering was 795,072,196, and the number of clean bases obtained from a total of six pools was 112.00 Gb, with clean reads ranging from 83,760,834 to 161,211,222 for a single pool, which indicated that the sequencing data were available for the subsequent analysis. At least 99.78% of the captured sequence fragments could be aligned to the CS RefSeq v1.0 by IWGSC, and the mean sequencing depth varied from 28.19 to 61.75×, with the exome region occupying more than 77.43% in each pool. These results indicated that the BSE-Seq assays among the pools were efficient in the present study. After SNP/InDel calling with BCFtools, a total of 3,980,637 SNPs and 231,018 InDels were obtained within all the six pools, of which 4,425,722 SNPs and 199,018 InDels were inconsistent with the reference sequence (Supplementary Table 4).

**FIGURE 4:**
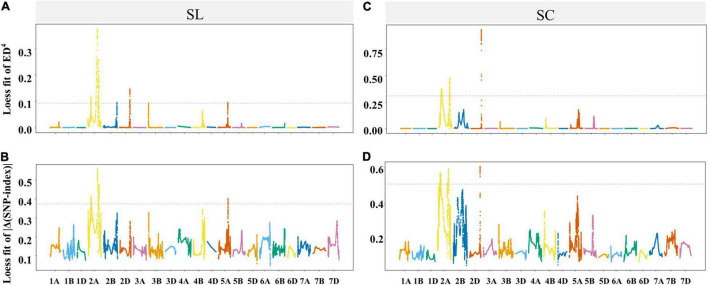
Locally weighted scatterplot smoothing (LOESS) fitting Manhattan for spikelet compactness (SC) and spike length (SL). Panels **(A–D)** were the LOESS fits of ED4 and | Δ(SNP-index)| for SL and SC, respectively. The cutoff of the two methods for SL and SC are shown by the dotted lines, and the threshold values for **(A–D)** were 0.0935, 0.3734, 0.3095, and 0.5107, respectively.

The genomic regions for SC were discovered on the chromosomes 2A and 2D based on the ED and SNP-index methods (Figure 4 and Supplementary Table 5). In addition, the genomic regions for SL were detected on the chromosomes 2A, 2B, 2D, 3B and 5A by ED method, and only on 2A by SNP-index method (Figure 4 and Supplementary Table 5). When two methods were taken into account at the same time by IWGSC RefSeq v2.1, two genomic regions associated with SL were identified in the regions of physical interval 182.16–194.35 and 581.7–660.76 Mb on chromosome 2A with 220 SNPs and 737 SNPs, and three genomic regions associated with SC were identified in the regions of 142.57–206.6 and 620.87–656.65 on chromosome 2A and 603.08–606.63 Mb on chromosome 2D with 602, 219, and 199 SNPs, respectively (Figure 4 and Supplementary Table 5). To investigate the polymorphism between two parents, a total of 2,463 SNP variant sites were discovered in the interesting regions of the chromosomes 2A and 2D between BLM and CY20 based on the Wheat 660K SNP array assay (Supplementary Table 6).

### Genetic Map Construction and Quantitative Trait Loci Identification

**FIGURE 5:**
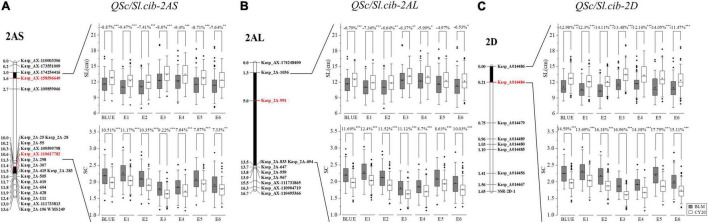
Genetic maps of three major QTL, *QSc/Sl.cib-2AS*, *QSc/Sl.cib-2AL*, and *QSc/Sl.cib-2D*, and their effects on spike compactness (SC) and spike length (SL) in BC20 populations. **(A)** Integrated genetic map containing the marker *Kasp_AX-158596649* and *Kasp_AX-110617782* and the effect of *QSc/Sl.cib-2AS* on SC and SL in the BC20 population. **(B)** Integrated genetic map containing the marker *Kasp_2A-991* and the effect of *QSc/Sl.cib-2AL* on SC and SL in the BC20 population. **(C)** Integrated genetic map containing the marker *Kasp_A014484* and the effect of *QSc/Sl.cib-2D* on SC and SL in the BC20 population. BLM and CY20 indicate the lines with the homozygous alleles from BLM and CY20, respectively. *, **, and *** represent significance at *P* < 0.05, *P* < 0.01, and *P* < 0.001, respectively.

To confirm the preliminarily identified genomic regions responsible for SC and SL, SNPs and InDels in the target regions were converted into KASP and SSR markers to construct the genetic map. In total, 40 KASP markers and two SSR markers were used for the construction of the genetic maps by genotyping 182 lines of the BC20 population (Supplementary Table 7). The resulting linkage maps of the interesting regions on chromosomes 2AS, 2AL, and 2D spanned 13.6, 16.7, and 1.65 cM in length, and contained 21 KASP and one SSR markers, 11 KASP markers, eight KASP and one SSR markers, respectively (Figure 5 and Supplementary Table 7). The phenotypic data of SC and SL evaluated in the six environments and their corresponding BLUE datasets were used for QTL mapping, and the BLUE datasets were treated as an additional environment. A total of six QTL, three for SC and three for SL, were repeatedly detected in at least four environments and the BLUE datasets, indicating that they were environmentally stable (Table 2).

**TABLE 2:** Quantitative trait loci (QTL) for spike compactness (SC) and spike length (SL) identified from the candidate region using BSE-Seq in different environments and the BLUE datasets.

QTL	Environment	chromosome	Genetic Interval (cM)	Flanking marker	LOD	PVE (%)	Add
*QSl*.*cib-2AS*	BLUE	2AS	0.98–1.55	Kasp_AX-174254416–Kasp_AX-158596649	3.76	9.08	−0.54
	E1	2AS	0.98–1.55	Kasp_AX-174254416–Kasp_AX-158596649	4.01	9.69	−0.56
	E3	2AS	0.98–1.55	Kasp_AX-174254416–Kasp_AX-158596649	3.27	7.93	−0.56
	E4	2AS	0.98–1.55	Kasp_AX-174254416–Kasp_AX-158596649	3.2	7.95	−0.6
	E5	2AS	0.98–1.55	Kasp_AX-174254416–Kasp_AX-158596649	2.5	6.16	−0.53
*QSc.cib-2AS*	BLUE	2AS	10.6–11.32	Kasp_AX-110617782–Kasp_2A-298	5.16	12.66	0.1
	E1	2AS	10.6–11.32	Kasp_AX-110617782–Kasp_2A-298	5.34	13.24	0.11
	E2	2AS	10.6–11.32	Kasp_AX-110617782–Kasp_2A-298	4.54	11.13	0.1
	E3	2AS	10.6–11.32	Kasp_AX-110617782–Kasp_2A-298	3.92	10.36	0.08
	E4	2AS	10.6–11.32	Kasp_AX-110617782–Kasp_2A-298	2.58	7	0.07
	E5	2AS	0.98–1.55	Kasp_AX-174254416–Kasp_AX-158596649	3.76	9.12	0.09
	E6	2AS	0.98–1.55	Kasp_AX-174254416–Kasp_AX-158596649	3.67	9.36	0.1
*QSl*.*cib-2AL*	BLUE	2AL	5.03–13.53	Kasp_2A-991–Kasp_2A-833	5.12	12.53	−0.64
	E1	2AL	5.03–13.53	Kasp_2A-991–Kasp_2A-833	4.86	11.75	−0.62
	E2	2AL	5.03–13.53	Kasp_2A-991–Kasp_2A-833	3.61	8.88	−0.55
	E3	2AL	5.03–13.53	Kasp_2A-991–Kasp_2A-833	4.83	12.09	−0.68
	E4	2AL	5.03–13.53	Kasp_2A-991–Kasp_2A-833	3.95	10.26	−0.68
	E6	2AL	5.03–13.53	Kasp_2A-991–Kasp_2A-833	2.85	7.37	−0.6
*QSc.cib-2AL*	BLUE	2AL	1.33–5.03	Kasp_2A-1036–Kasp_2A-991	6.38	14.5	0.12
	E1	2AL	1.33–5.03	Kasp_2A-1036–Kasp_2A-991	6.71	15.26	0.13
	E2	2AL	1.33–5.03	Kasp_2A-1036–Kasp_2A-991	5.37	12.47	0.11
	E3	2AL	1.33–5.03	Kasp_2A-1036–Kasp_2A-991	5.28	12.32	0.09
	E4	2AL	5.03–13.53	Kasp_2A-991–Kasp_2A-833	3.56	9.32	0.09
	E5	2AL	5.03–13.53	Kasp_2A-991–Kasp_2A-833	4.26	10.47	0.11
	E6	2AL	5.03–13.53	Kasp_2A-991–Kasp_2A-833	4.97	12.51	0.12
*QSl*.*cib-2D*	BLUE	2D	0–0.21	Kasp_A014486–Kasp_A014484	9.49	21.35	−0.83
	E1	2D	0–0.21	Kasp_A014486–Kasp_A014484	7.62	17.63	−0.74
	E2	2D	0–0.21	Kasp_A014486–Kasp_A014484	9.95	22.26	−0.85
	E3	2D	0–0.21	Kasp_A014486–Kasp_A014484	9.53	21.43	−0.91
	E4	2D	0–0.21	Kasp_A014486–Kasp_A014484	6.02	14.42	−0.81
	E5	2D	0–0.21	Kasp_A014486–Kasp_A014484	7.6	17.57	−0.89
	E6	2D	0–0.21	Kasp_A014486–Kasp_A014484	4.51	11.36	−0.72
*QSc.cib-2D*	BLUE	2D	0–0.21	Kasp_A014486–Kasp_A014484	10.6	23.52	0.14
	E1	2D	0–0.21	Kasp_A014486–Kasp_A014484	8.42	19.28	0.14
	E2	2D	0–0.21	Kasp_A014486–Kasp_A014484	11.88	25.97	0.15
	E3	2D	0–0.21	Kasp_A014486–Kasp_A014484	12.84	27.74	0.13
	E5	2D	0–0.21	Kasp_A014486–Kasp_A014484	13.22	28.56	0.17
	E6	2D	0–0.21	Kasp_A014486–Kasp_A014484	7.61	18.44	0.13

*PVE, phenotypic variation explained; LOD, logarithm of the odd; Add, additive effect (positive values indicate that alleles from BLM are increasing the trait scores, and negative values indicate that alleles from CY20 are increasing the trait scores); BLUE, best linear unbiased estimation.*

Three QTL, *QSc.cib-2AS*, *QSc.cib-2AL*, and *QSc.cib-2D*, were detected for SC. *QSc.cib-2AS* was detected in all the environments and the BLUE dataset showed the LOD values ranging from 2.58 to 5.34 and explained 7.00–13.24% of the phenotypic variance. *QSc.cib-2AL* was also detected in the six environments and the BLUE dataset and explained 9.32–15.26% of the phenotypic variance, together with the LOD values ranging from 3.56 to 6.71. *QSc.cib-2D* was detected in the BLUE dataset and five environments with LOD values in the range of 7.61–13.22, which explained 18.44–28.56% of the phenotypic variance. All the favorable alleles of the three loci were contributed by BLM (Table 2).

Three QTL, *QSl.cib-2AS*, *QSl.cib-2AL*, and *QSl.cib-2D*, were detected for SL in the BC20 population. *QSl.cib-2AS* was stably detected in four environments and the BLUE dataset, and it explained 6.16–9.08% of the phenotypic variance with LOD values ranging from 2.5 to 4.01. *QSl.cib-2AL* was detected in five environments and the BLUE dataset, with the LOD values varying from 2.85 to 5.12 and explaining 7.37–12.53% of the phenotypic variance. *QSl.cib-2D* was detected in all the environments and the BLUE dataset with the phenotypic variance explained (PVE) ranging from 11.36% to 22.26% and the LOD values varying from 4.51 to 9.95. CY20 contributed all the favorable alleles of the three loci.

Moreover, several QTL for PHT, TGW, and GL were detected on chromosome 2A (Supplementary Table 8). Among them, *QPht.cib-2AS and QPht.cib-2AL* were detected in at least three environments and explained 7.06–10.16% and 8.29–10.80% of the phenotypic variance, respectively.

Although the intervals of *QSl.cib-2AS* and *QSc.cib-2AS* on the chromosome arm 2AS showed different genetic positions, analyses of the corresponding physical locations revealed that the genomic region of *QSc.cib-2AS* contained the region of *QSl.cib-2AS*. Moreover, two QTL *QSl.cib-2AL* and *QSc.cib-2AL* on chromosome arm 2AL shared the same flanking marker *Kasp_2A-991*. In addition, the two QTL (*QSl.cib-2D* and *QSc.cib-2D*) on chromosome 2D had the same flanking markers of *KASP_A014486* and *KASP_A014484* with identical genetic intervals and physical regions (Figure 5 and Table 2). Thus, the three loci were temporarily designed as *QSc/Sl.cib-2AS*, *QSc/Sl.cib-2AL*, and *QSc/Sl.cib-2D*.

### Effects of *QSc/Sl.cib-2AS*, *QSc/Sl.cib-2AL*, and *QSc/Sl.cib-2D* on Spike Compactness and Length in BC20 Population

For *QSc/Sl.cib-2AS* and *QSc/Sl.cib-2D*, significant differences (*P* < 0.001 in all environments and BLUE except E6, *P* < 0.01 only in E6) in SC and SL were detected between the two groups among all the environments and the BLUE datasets. For *QSc/Sl.cib-2AL*, significant differences (*P* < 0.001 in BLUE and E1, *P* < 0.01 in E2 and E3, and *P* < 0.05 in E4 and E6) in SL were detected between the two groups, and no significant difference in SL was observed in E5, with the spike length increased by 4.97% of the group of lines with homozygous alleles from BLM; however, strong differences (*P* < 0.001) in SC were detected in all the environments and the BLUE dataset (Figure 5).

**FIGURE 6:**
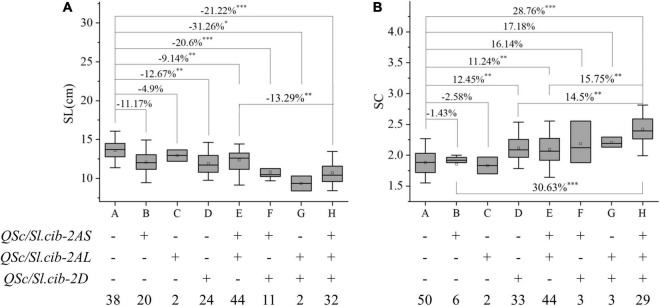
Additive effects of three QTL on spike compactness (SC, **B**) and spike length (SL, **A**), respectively. Symbols + and – represent lines with and without the positive alleles for the target quantitative trait loci (QTL) based on the flanking marker of the corresponding QTL, respectively. *, ^**^, and ^***^ represent significance at *P* < 0.05, *P* < 0.01, and *P* < 0.001, respectively.

Since the effects of *QSc/Sl.cib-2AS*, *QSc/Sl.cib-2AL*, and *QSc/Sl.cib-2D* on SC and SL could be detected at the same time, their additive effects on the corresponding traits were analyzed. Based on the genotyping data of tightly linked markers of three QTL, the lines from the BC20 population were divided into the following eight groups: A, lines carrying the alleles from CY20 at all the three loci; B, lines only carrying the alleles from BLM at *QSc/Sl.cib-2AS*; C, lines only carrying the alleles from BLM at *QSc/Sl.cib-2AL*; D, lines only carrying the alleles from BLM at *QSc/Sl.cib-2D*; E, lines carrying the alleles from BLM at *QSc/Sl.cib-2AS* and *QSc/Sl.cib-2AL*; F, lines carrying the alleles from BLM at *QSc/Sl.cib-2AS* and *QSc/Sl.cib-2D*; G, lines carrying the alleles from BLM at *QSc/Sl.cib-2AL* and *QSc/Sl.cib-2D*; H, lines carrying the alleles from BLM at *QSc/Sl.cib-2AS, QSc/Sl.cib-2AL* and *QSc/Sl.cib-2D* (Figure 6). Compared with the lines carrying the alleles from CY20 at *QSc/Sl.cib-2AS*, *QSc/Sl.cib-2AL* and *QSc/Sl.cib-2D*, the lines carrying alleles of BLM at one of the two loci *QSc/Sl.cib-2AS* and *QSc/Sl.cib-2D*, thus group B and D, decreased SL by 11.17% and 12.67%, respectively, and the loci *QSc/Sl.cib-2D* alone increased SC by 12.45% (*P* < 0.01). The lines with homozygous alleles of BLM at two of the three loci, for example group E and F, significantly reduced SL by 9.14% and 20.6%, respectively, and simultaneously group E significantly increased SC by 11.24%. Interestingly, the combination of lines with alleles from BLM at all three loci contributed to a 21.22% decline in SL and a 28.76% increase in SC with *P* < 0.001, which were higher than the combination lines with one or two loci from BLM (Figure 6).

### Effects of *QSc/Sl.cib-2AS*, *QSc/Sl.cib-2AL*, and *QSc/Sl.cib-2D* on Yield-Related Traits in BC20 Population

**FIGURE 7:**
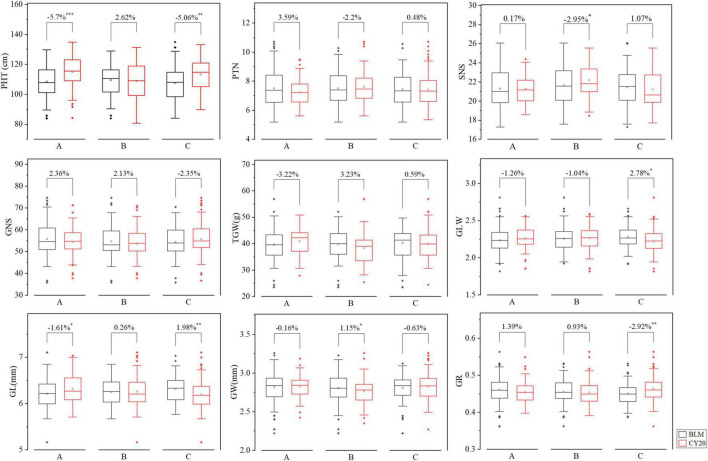
Effects of *QSc/Sl.cib-2AS* (A), *QSc/Sl.cib-2AL* (B), and *QSc/Sl.cib-2D* (C) on plant height (PHT), productive tiller number (PTN), spikelet number per spike (SNS), grain number per spike (GNS), thousand grain weight (TGW), GL/GW (GLW) ratio, grain length (GL), grain width (GW), and grain roundness (GR) in the BC20 population. BLM and CY20 represent lines with the alleles from BLM and CY20, respectively. *, **, and *** represent significance at *P* < 0.05, *P* < 0.01, and *P* < 0.001, respectively.

The BLUE datasets were further used to analyze the effects of *QSc/Sl.cib-2AS*, *QSc/Sl.cib-2AL*, and *QSc/Sl.cib-2D* on other yield-related traits in the mapping populations. For *QSc/Sl.cib-2AS*, the lines with homozygous alleles from BLM had lower PHT (*P* < 0.001) and GL (*P* < 0.05) when compared to the lines carrying the alleles from CY20 (Figure 7). At the locus *QSc/Sl.cib-2AL*, the lines with alleles from BLM had lower SNS and higher GW (*P* < 0.05) when compared to the lines carrying the alleles from CY20 (Figure 7). For *QSc/Sl.cib-2D*, lines possessing the alleles from BLM showed a significant decrease in PHT and GR (*P* < 0.01) and a strong increase in GLW (*P* < 0.05) and GL (*P* < 0.01). No significant effects were observed on the traits of PTN, GNS, and TGW between the two groups in the BC20 population (Figure 7). Moreover, no significant difference was detected in SNS, GLW, GW, and GR between the two groups at *QSc/Sl.cib-2AS*, no significant difference in PTN, GLW, GL and GR at *QSc/Sl.cib-2AL*, and no significant effects in SNS and GR at *QSc/Sl.cib-2D* (Figure 7). In addition, for *QSc/Sl.cib-2AS* and *QSc/Sl.cib-2D*, lines carrying alleles from BLM simultaneously and significantly reduced PHT, but had opposite effects on GL (Figure 7).

### Potential Candidate Genes for *QSc/Sl.cib-2AS*, *QSc/Sl.cib-2AL*, and *QSc/Sl.cib-2D*

**FIGURE 8:**
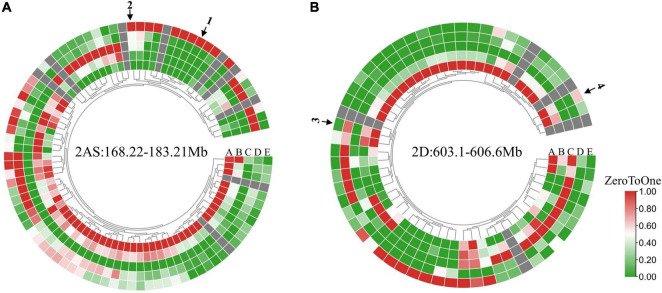
Expression pattern of genes within the *QSc/Sl.cib-2AS* **(A)** and *QSc/Sl.cib-2D* **(B)** intervals. Expression data originated from the WheatOmics GeneExpression (http://202.194.139.32/expression/index.html). The arrows 1, 2, 3, and 4 represent the predicted candidate genes *TraesCS2A03G0410600*, *TraesCS2A03G0422300*, *TraesCS2D03G1129300*, and *TraesCS2D03G1131500*. A, B, C, D, and E **(A,B)** represent root, stem, leaf, grain, and spike, respectively.

According to the CS reference genome v2.1 (Zhu et al., 2021), there were 88, 368 and 60 annotated high-confidence genes within the candidate intervals of *QSc/Sl.cib-2AS*, *QSc/Sl.cib-2AL*, and *QSc/Sl.cib-2D*, respectively (Figure 8, Supplementary Figure 1, and Supplementary Tables 9–11). Analysis of the spatiotemporal expression patterns (Borrill et al., 2016; Ramirez-Gonzalez et al., 2018) showed that there were 13, 45 and nine genes in *QSc/Sl.cib-2AS*, *QSc/Sl.cib-2AL* and *QSc/Sl.cib-2D* regions that were highly or specifically expressed in spike, which might be probably involved in spike growth and development (Figure 8 and Supplementary Tables 9–11). Combining the annotations of homolog gene functions in rice and *Arabidopsis*, four potential candidate genes, *TraesCS2A03G0410600* and *TraesCS2A03G0422300* for *QSc/Sl.cib-2AS*, and *TraesCS2D03G1129300* and *TraesCS2D03G1131500* for *QSc/Sl.cib-2D*, were likely associated with the spike compactness and length in the study (Supplementary Tables 9–11). Based on the BSE-Seq data, nonsynonymous SNPs were detected in the coding region of *TraesCS2A03G0410600*, *TraesCS2A03G0422300*, *TraesCS2D03G1129300* and *TraesCS2D03G1131500* (Supplementary Table 12).

## Discussion

### Comparison of *QSc/Sl.cib-2AS*, *QSc/Sl.cib-2AL*, and *QSc/Sl.cib-2D* to Those Reported in Previous Studies

In the present study, six QTL for SC and SL were identified in at least four environments and in the BLUE datasets, indicating that they were environmentally stable (Table 2). We compared their physical intervals with those detected previously. Only major and stable QTL have potential use in marker-assisted selection (MAS) in breeding programs. Therefore, the subsequent discussion was based on the major and stable QTL reported previously.

*QSc/Sl.cib-2AS*, comprising *QSc.cib-2AS* and *QSl.cib-2AS* loci, was physically co-localized in the interval of 167.65–183.91 Mb on the short arm of chromosome 2A (Table 2). Comparison analysis revealed that there were 13 loci for SC or/and SL on the chromosome 2AS identified previously, but among them, no major and sable QTL was overlapped or close to *QSc/Sl.cib-2AS* in the present study (Yao et al., 2009; Zhang et al., 2009; Li et al., 2016, 2019b, 2021a; Fu et al., 2017; Liu et al., 2018, 2019) (Supplementary Table 13). *QSc/Sl.cib-2AS* showed a significant effect on PHT and GR, but no effect on SNS in our study. Thus, *QSc/Sl.cib-2AS* was likely a novel QTL simultaneously for both SC and SL.

Thus, *QSc/Sl.cib- 2AL* was probably a novel QTL for SC and SL. *QSc/Sl.cib-2AL*, containing *QSc.cib-2AL* and *QSl.cib-2AL* loci, was identified within the interval of 575.17–620.94 Mb on the long arm of the chromosome 2A (Figure 5, Table 2, and Supplementary Table 7). There were 13 QTL for SC and/or SL reported previously. Among them, no major and stable locus for spike compactness and length was reported (Supplementary Table 13) (Yao et al., 2009; Faris et al., 2014; Lee et al., 2014; Gao et al., 2015; Li et al., 2019b, 2021a; Sheoran et al., 2019; Chen et al., 2020b). *QSc/Sl.cib-2AL* had a significant (*P* < 0.05) effect on reducing the spikelet number but had no effect on the plant height, which was different to *QSc/Sl.cib-2AS* and *QSc/Sl.cib-2AD*.

*QSc/Sl.cib-2D*, including *QSc.cib-2D* and *QSl.cib-2D*, was identified in the interval of 603.08–606.63 Mb on the long arm of the chromosome 2D. Based on BSE-Seq and Wheat 660K SNP array assay results, no extra SNP and InDel sites on the chromosomal fragments of each 50 Mb upstream and downstream of the region 603.08–606.63 Mb could be converted into SSR or KASP markers. According to the results from BSE-Seq and genetic linkage analysis, a major and stable QTL for SC and SL located nearby the marker *Kasp_A014484* is reliable. However, this interval (603.08–606.63 Mb) may be incomplete and unreliable for QTL mapping and potential gene prediction for SC and SL, and have no influence on the results of SC and SL interactions and effects on the corresponding trait and other agronomic traits (Figures 4, 5 and Supplementary Tables 5, 7). Comparative analysis revealed that there were six QTL for SC reported previously were overlapped with this region (Supplementary Table 13). For instance, *QSD.SAU-2N.2* and *QSc.cau-2D.1* for SC were located in the interval of 405.73–654.34 and 423.69–654.34 Mb, which were much larger than *QSc/Sl.cib-2D* (Gao et al., 2016b; Zhai et al., 2016). *Qsd.sac-2D*, *QSd.sau-2SY-2D*, *QSd.sicau-2D.3* and the marker *IWB26541* for SC reported previously located within 588.68–612.82, 602.76–610.04, 605.12–609.88 and 611.14 Mb were overlapped and covered the interval in the present study (Luo et al., 2016; Liu et al., 2018, 2019; You et al., 2021). Among them, QTL for SL and SNS were detected at the locus *QSc.cau-2D.1* in the interval of 423.69–654.34 Mb, indicating that it was a multiple effect locus (Zhai et al., 2016). In addition, previous studies revealed that *QSd.sau-2SY-2D* exerted pleiotropic effects on SL, SNS and TKW (You et al., 2021). Moreover, *QSc/Sl.cib-2D* had pleiotropic effects on PHT, GLW, GL and GR (Figure 7). Thus, *QSc/Sl.cib-2D* was a major and stable QTL overlapped with those reported previously.

### Pyramiding of *QSc/Sl.cib-2AS*, *QSc/Sl.cib-2AL* and *QSc/Sl.cib-2D* for Trait Improvement

Integrating multiple favorable alleles into a genetic background was considered to be an effective strategy to optimize traits for yield increasing in wheat (Fan et al., 2019; Li et al., 2021a,b; Tu et al., 2021; You et al., 2021). *QSc/Sl.cib-2AS* or *QSc/Sl.cib-2AL* alone had no significant effect on SC and SL, but *QSc/Sl.cib-2D* alone could strongly decrease SL by 12.76% and increase SC by 12.45%, respectively (Figure 6). The combination of two or three favorable alleles had significant additive effects on SC and SL. Interestingly, compared with single *QSc/Sl.cib-2AS* or *QSc/Sl.cib-2AL* alone, the combination of *QSc/Sl.cib-2AS* and *QSc/Sl.cib-2AL* and any combination containing *QSc/Sl.cib-2D* could significantly increase SL. Therefore, *QSc/Sl.cib-2D* showed the strongest effect on SC and SL in the present study (Figure 6). The significant aggregation effect indicated that the pyramiding of no matter what two or three loci had the potential to optimize the ideal architecture of spike morphology.

### Potential Candidate Genes for *QSc/Sl.cib-2AS* and *QSc/Sl.cib-2D*

On the CS genome, there are 88 and 60 annotated high-confidence genes in the physical interval of *QSc/Sl.cib-2AS* and *QSc/Sl.cib-2D*. Based on the gene annotation, expression analysis and orthologous gene analysis, two genes *TraesCS2A03G0410600* and *TraesCS2A03G0422300* for *QSc/Sl.cib-2AS*, and two genes *TraesCS2D03G1129300* and *TraesCS2D03G1131500* for *QSc/Sl.cib-2D* were found to be likely involved in the growth and development of spike (Figure 8 and Supplementary Tables 9, 11).

*TraesCS2A03G0410600*, expressed specifically in spike, encodes a member of the plant-specific YABBY transcription factor family, which was reported to play important roles in the formation and development of reproductive organs in plants (Zhang et al., 2020). *TraesCS2A03G0422300*, encoding a putative NAC domain-containing protein, is an ortholog of *AT1G61110*, which was reported to be involved in the embryo development and expressed during the growth and developmental stages of flowering, petal differentiation and expansion stage in *Arabidopsis* (Sánchez-Montesino et al., 2019). *TraesCS2D03G1129300* was annotated to *APETALA 2* (*AP2*)-like ethylene-responsive transcription factor that was reported to be the key factors in inflorescence branching and rice domestication (Harrop et al., 2019). The famous gene *Q* (*AP2L5*), belonging to the AP2-like protein family, and its related paralog *AP2L2* were reported to play critical and redundant roles in the specification of axillary floral meristems and lemma identity in wheat (Debernardi et al., 2020). *TraesCS2D03G1131500* was annotated to the Jasmonic acid (JA) signaling repressor to regulate spikelet development (Wang et al., 2008). The sequence analysis revealed that *TraesCS2A03G0410600*, *TraesCS2A03G0422300*, *TraesCS2D03G1129300* and *TraesCS2D03G1131500* have nonsynonymous SNPs in the coding region (Supplementary Table 12). These four candidate genes for *QSc/Sl.cib-2AS* and *QSc/Sl.cib-2D* would be the focus of fine-mapping analyses in the future.

## Data Availability Statement

The datasets presented in this study can be found in online repositories. The names of the repository/repositories and accession number(s) can be found in the article/Supplementary Material.

## Author Contributions

QY performed all the experiments and subsequent analysis of all the available data including phenotyping and population genotyping, and wrote the manuscript. TW, PG, and BF designed the experiments, guided the entire study, and revised the manuscript. ZX constructed the mapping population and assisted in field trials. XF helped in ordering the reagents. TW and BF held primary responsibility for the final content. All authors read and reviewed the manuscript.

## Conflict of Interest

The authors declare that the research was conducted in the absence of any commercial or financial relationships that could be construed as a potential conflict of interest.

## Publisher’s Note

All claims expressed in this article are solely those of the authors and do not necessarily represent those of their affiliated organizations, or those of the publisher, the editors and the reviewers. Any product that may be evaluated in this article, or claim that may be made by its manufacturer, is not guaranteed or endorsed by the publisher.

## Footnotes

1
http://www.isbreeding.net/software/

2
http://www.whbioacme.com

3
http://202.194.139.32/

4
https://galaxy.triticeaetoolbox.org/

5
http://wheat.pw.usda.gov/ggpages/wgc/98/Intro.htm

6
http://www.uniprot.org/

## Supplementary Material

Supplementary Figure 1 Expression pattern of genes within the *QSc/Sl.cib-2AL* interval. Expression data originated from the WheatOmics GeneExpression (http://202.194.139.32/expression/index.html). A, B, C, D, and E **(A,B)** represent root, stem, leaf, grain, and spike, respectively.
