# Supplementary figures and images for: Genetic Dissection of Three Major Quantitative Trait Loci for Spike Compactness and Length in Bread Wheat (Triticum aestivum L.)
Source: Front Plant Sci. 2022 May 23;13:882655. doi: 10.3389/fpls.2022.882655 (PMC9168683; doi:10.3389/fpls.2022.882655)

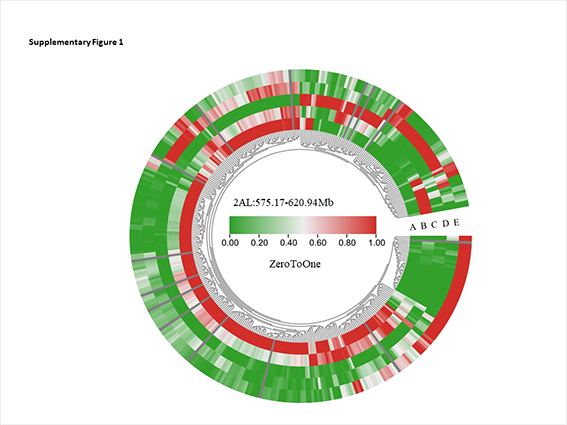

Supplement: Supplementary Figure 1 — Expression pattern of genes within the QSc/Sl.cib-2AL interval. Expression data originated from the WheatOmics GeneExpression (http://202.194.139.32/expression/index.html). A, B, C, D, and E (A,B) represent root, stem, leaf, grain, and spike, respectively. [file Image_1.TIF]
